# Inborn errors of amino acid metabolism – from underlying pathophysiology to therapeutic advances

**DOI:** 10.1242/dmm.050233

**Published:** 2023-11-23

**Authors:** Shira G. Ziegler, Jiyoung Kim, Jeffrey T. Ehmsen, Hilary J. Vernon

**Affiliations:** ^1^ Department of Genetic Medicine, Johns Hopkins University School of Medicine, Baltimore, MD 21205, USA; ^2^ Department of Internal Medicine, Johns Hopkins University School of Medicine, Baltimore, MD 21205, USA

**Keywords:** Amino acids, Inborn errors of metabolism, Newborn screen

## Abstract

Amino acids are organic molecules that serve as basic substrates for protein synthesis and have additional key roles in a diverse array of cellular functions, including cell signaling, gene expression, energy production and molecular biosynthesis. Genetic defects in the synthesis, catabolism or transport of amino acids underlie a diverse class of diseases known as inborn errors of amino acid metabolism. Individually, these disorders are rare, but collectively, they represent an important group of potentially treatable disorders. In this Clinical Puzzle, we discuss the pathophysiology, clinical features and management of three disorders that showcase the diverse clinical presentations of disorders of amino acid metabolism: phenylketonuria, lysinuric protein intolerance and homocystinuria due to cystathionine β-synthase (CBS) deficiency. Understanding the biochemical perturbations caused by defects in amino acid metabolism will contribute to ongoing development of diagnostic and management strategies aimed at improving the morbidity and mortality associated with this diverse group of disorders.

## Teaser

**Summary:** This Clinical Puzzle summarizes the pathophysiology, clinical presentation and treatment option of three disorders of amino acid metabolism, namely, phenylketonuria, lysinuric protein intolerance and homocystinuria due to cystathionine β-synthase deficiency.

## Introduction

Amino acids are organic molecules that include basic amino and carboxylic acid groups and serve as the building blocks of proteins. Of the twenty proteinogenic amino acids, humans can endogenously synthesize eleven, either from other amino acids or from other metabolic precursors. The other nine – histidine, isoleucine, leucine, lysine, methionine, phenylalanine, threonine, tryptophan and valine – are essential, as they cannot be endogenously synthesized and therefore must be obtained from the diet (Broer and Broer, 2017).

In addition to their role as substrates for protein synthesis, amino acids play essential roles in cell signaling, regulation of gene expression, energy production, and as molecular substrates for other key metabolites such as biogenic amines, purines and pyrimidines (Broer and Broer, 2017; Tetrick and Odle, 2020; Wu, 2009). Thus, abnormalities in amino acid biosynthesis and degradation have the potential to underlie complex multisystem health problems.

Genetic defects in the biosynthesis, degradation or transport of amino acids lead to diverse inborn errors of amino acid metabolism, including disorders of branched-chain amino acid metabolism, glycine metabolism, aromatic amino acid metabolism, sulfated amino acid metabolism and others (Table 1). Individually, inborn errors of amino acid metabolism are rare, but collectively, they represent an important class of potentially treatable disorders. Other classes of inborn errors of metabolism, such as urea cycle defects and organic acidemias, also overlap with disorders of amino acid metabolism in their biochemical basis and/or clinical presentations, and will not be addressed in this review.

**Table 1.**
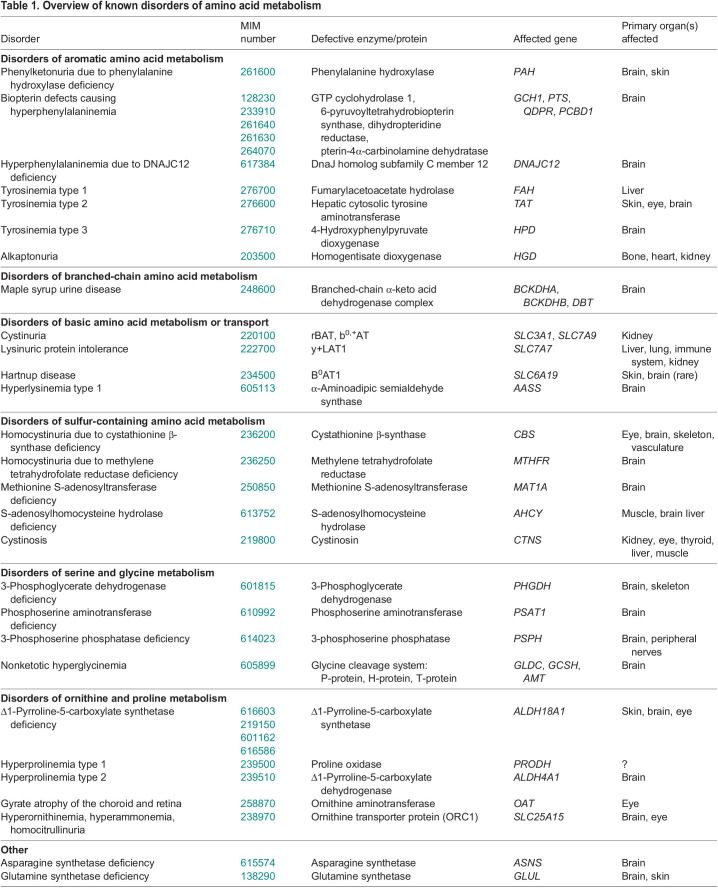
Overview of known disorders of amino acid metabolism

Despite their diversity, inborn errors of amino acid metabolism share several common features and some are included in newborn screening (NBS) panels, with the goal of early detection and immediate initiation of treatment (Waters et al., 2018). For those not detected by or included in NBS, early recognition of clinical and laboratory features in symptomatic patients is critical for the timely initiation of appropriate and disease-specific management. The classical approach to treating genetic disorders of amino acid metabolism focuses on correcting biochemical defects by reducing upstream metabolite accumulation, replacing abnormal enzymes and/or cofactors and providing deficient downstream products (Vernon and Manoli, 2021). However, this treatment approach is less straightforward in biochemically complex amino acid disorders in which upstream or downstream toxic metabolites are not completely understood or cannot be modified. More recently, enzyme and gene replacement approaches are being used or developed, which may ultimately bypass the necessity to recognize and correct all of the individual metabolic excursions particular to each disorder.

**Fig. 1.**
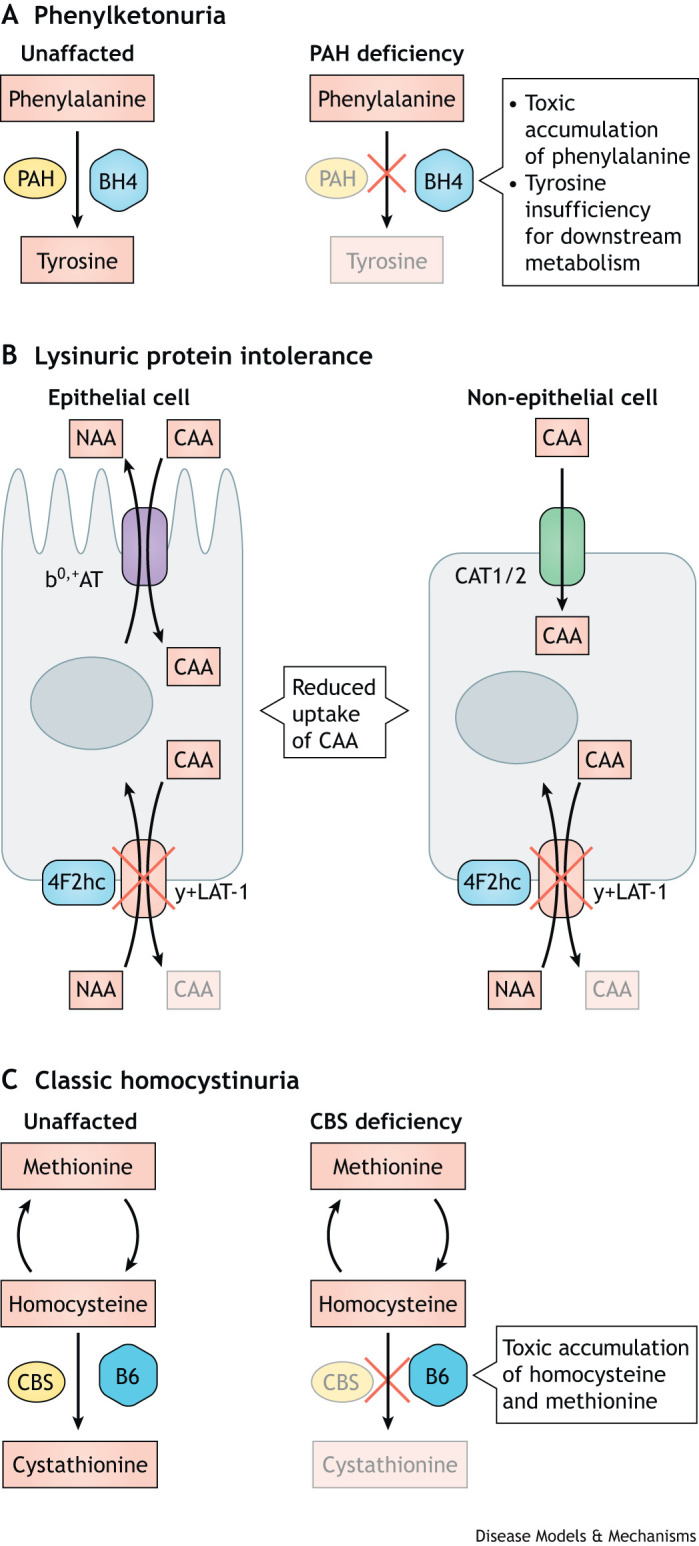
**Three genetic disorders of amino acid metabolism.** (A) Phenylketonuria is caused by defects in phenylalanine hydroxylase (PAH) that result in the inability to metabolize phenylalanine to tyrosine, leading to toxic accumulation of phenylalanine. PAH activity depends on the cofactor tetrahydrobiopterin (BH4), meaning that certain PAH deficiencies can be ameliorated by increasing the availability of BH4. (B) The cationic amino acid (CAA) transporter complex y(+) L-type amino acid transporter 1 (y+LAT1)-4F2hc is responsible for importing ornithine, arginine and lysine and for exporting various neutral amino acids (NAAs), whereas the b^0,+^ amino acid transporter (b^0,+^AT) imports CAAs and exports NAAs in epithelial cells, and the CAT1 and CAT2 transporters (encoded by *SLC7A1* and *SLC7A2*, respectively) import CAAs in non-epithelial cells. Lysinuric protein intolerance is caused by defects in the y+LAT1 subunit of y+LAT1-4F2hc, which results in insufficient CAA transport in epithelial and non-epithelial cells, leading to malabsorption and secondary defects in the urea cycle. (C) Classical homocystinuria is caused by a defect in cystathionine β-synthase (CBS), which metabolizes homocysteine to cystathione with vitamin B6 as cofactor, leading to toxic accumulation of methionine and homocysteine.

Here, we will discuss the pathophysiology, diagnosis, clinical features and management of three autosomal recessive disorders of amino acid metabolism: phenylketonuria (PKU), lysinuric protein intolerance (LPI) and homocystinuria due to cystathionine β-synthase (CBS) deficiency (Fig. 1). PKU, LPI and homocystinuria showcase the diverse clinical presentations of disorders of amino acid metabolism: PKU as a disorder with cerebral intoxication, LPI as a disorder with acute metabolic decompensation events and unique pulmonary and immune-related features, and homocystinuria as a disorder with prominent hematologic and connective tissue implications. In addition to their strikingly different clinical presentations, these disorders also represent different prevalence, treatment paradigms and biochemical effects.

## Phenylketonuria

### Clinical features and epidemiology

Phenylketonuria (PKU) is a canonical example of an amino acid disorder of cerebral intoxication: in this disease, the intoxication is by phenylalanine. Depending on the concentration of blood phenylalanine and residual phenylalanine hydroxylase (PAH) enzymatic activity, PAH deficiency (OMIM #261600) can present with a range of clinical phenotypes – from mild hyperphenylalaninemia to classic PKU (Regier and Greene, 1993). Lower residual PAH activity usually results in higher phenylalanine concentrations and a more severe clinical phenotype if left untreated (Himmelreich et al., 2018). PKU is the most common autosomal recessive Mendelian disorder of amino acid metabolism, estimated to affect 0.45 million individuals worldwide. Based on NBS data, the prevalence of PKU varies globally – ranging from one in 4500 in Italy to one in 125,000 in Japan, with an average prevalence of one in 10,000 (Hillert et al., 2020).

Infants with untreated persistent severe hyperphenylalaninemia, in which phenylalanine levels exceed 1200 µmol/l, usually present with varying degrees of epilepsy, irreversible intellectual disability, Parkinsonian-like features, musty body odor, eczema, and decreased skin and hair pigmentation (Regier and Greene, 1993). Patients with classic PKU, even when identified and treated from birth, can still have neuropsychological issues, including reduced attention span and executive function impairment, and are at high risk for anxiety and depression (Burton et al., 2013). Lapses in or discontinuation of dietary therapy (discussed in detail below) exacerbate these neuropsychological issues (Koch et al., 2002).

Affected individuals on an unrestricted diet who have phenylalanine levels between 600 and 1200 µmol/l are less likely to develop cognitive impairment, although they can have neuropsychological complications. Dietary management is strongly recommended. Individuals with phenylalanine levels less than 600 µmol/l on an unrestricted diet are at a relatively lower risk of developing intellectual, neurological or neuropsychological sequelae, and are usually only treated if phenylalanine levels are persistently above 360 µmol/l.

High phenylalanine levels in an untreated pregnant mother with PKU can also have severe consequences to a fetus. Termed maternal PKU syndrome, a fetus exposed to high maternal phenylalanine concentrations can develop intellectual disability, microcephaly and congenital malformations, including congenital heart defects, tracheoesophageal fistula and intrauterine growth restriction (Vockley et al., 2014).

### Pathophysiology

Defects in PAH result in the inability to metabolize phenylalanine to tyrosine (Fig. 1A), leading to accumulation of the phenylalanine substrate and lack of the tyrosine product, which is necessary for downstream production of neurotransmitters, conversion to thyroxine and melanin, and catabolism to acetoacetate and fumarate to be utilized as an energy source (van Spronsen et al., 2021).

Cell culture systems and mouse models have provided insights into the underlying pathophysiology of PAH deficiency. It has long been presumed that phenylalanine itself is neurotoxic given the clinical phenotype of patients with hyperphenylalaninemia, although the exact mechanism is unclear and is likely multifactorial. Studies have shown that phenylalanine inhibits neurite growth in culture and implicates the involvement of brain-derived neurotrophic factor (BDNF) (Li et al., 2010). This extends to humans: the complexity of dendritic branching and the number of synaptic connections are reduced in untreated individuals with PKU compared to those in unaffected individuals (Bauman and Kemper, 1982; Huttenlocher, 2000). There is also evidence that phenylalanine can assemble into fibrillar amyloid-like deposits – which resemble plaques seen in Alzheimer’s disease – in a PAH-deficient (PKU) mouse model and a single post-mortem affected human brain (Adler-Abramovich et al., 2012).

In addition to phenylalanine toxicity, the lack of downstream phenylalanine metabolites, including brain amines such as dopamine, norepinephrine and serotonin, also contributes to the disease process (Pascucci et al., 2002; Puglisi-Allegra et al., 2000). As dietary tyrosine supplementation alone does not prevent the neurocognitive features seen in individuals with PKU (Batshaw et al., 1981), it is hypothesized that elevated levels of phenylalanine in the blood compete with other amino acids for binding to the large amino acid transporter (LAT1; also known as SLC7A5) in the blood-brain barrier. This subsequently leads to high cerebral phenylalanine concentrations and low concentrations of neurotransmitter precursors. There is also growing evidence that a mitochondrial energy deficit and oxidative stress are key players in PKU pathophysiology (Dobrowolski et al., 2022; Ercal et al., 2002; Kienzle-Hagen et al., 2002). Data from rodent models – both PAH mutant mice and rats fed high-phenylalanine diets – in addition to *in vitro* studies consistently show alterations in mitochondrial function, ranging from disturbances in respiratory chain electron flow to increased apoptosis. These findings have primarily been attributed to the toxic effects of phenylalanine (summarized in Wyse et al., 2021).

### Diagnosis

PKU is usually identified during NBS based on the detection of hyperphenylalaninemia with tandem mass spectrometry on a dried blood spot (Box 1). Indeed, NBS was first developed to screen for classic PKU. Dr Robert Guthrie, a medical microbiologist in the 1960s, was the first to collect heel blood samples from newborns and implement a screening test to identify patients with PKU (Guthrie and Susi, 1963). In 1962, after screening only 800 newborns, the first case of PKU was identified (Guthrie and Susi, 1963). These early pursuits laid the foundation for NBS, which has now expanded to cover over 35 conditions in the USA (Levy, 2021b).

**Box 1. Case study**

Newborn screening results on an otherwise healthy 1-week-old infant showed an elevated blood phenylalanine level of 230 μmol/l (normal range <120 μmol/l). The infant was seen in a genetics clinic for follow-up testing by 2 weeks of age, and the results of this follow-up showed an elevated plasma phenylalanine level of 590 μmol/l with a low tyrosine level and an elevated phenylalanine-to-tyrosine ratio. Urine studies demonstrated normal biopterin intermediates. The infant was immediately started on a low-phenylalanine diet with a combination of natural breastmilk and phenylalanine-free formula. Blood phenylalanine levels remained normal on repeat testing 1 week after initiating dietary treatment. Molecular analysis demonstrated two pathogenic variants in the *PAH* gene in trans in this infant. With ongoing management and maintenance of appropriate blood phenylalanine levels, this infant is expected to have typical growth and development. As he gets older, other therapies, including BH4 co-factor and enzyme replacement therapies, may potentially be added to his treatment regimen.

A diagnosis of PKU is confirmed by both biochemical and molecular methods. The diagnosis is established when plasma phenylalanine concentration is persistently above 120 µmol/l, the patient exhibits an altered ratio of phenylalanine to tyrosine with normal tetrahydrobiopterin (BH4; the endogenous co-factor to PAH) metabolism and/or molecular testing finds biallelic pathogenic variants in the *PAH* gene. Worldwide, the three most common disease-causing variants in *PAH* are c.1222C>T (p.Arg408Trp), c.1066−11G>A (IVS10−11G>A) and c.782G>A (p.Arg261Gln) (Hillert et al., 2020).

Although the majority of hyperphenylalaninemia cases are caused by biallelic pathogenic variants in *PAH*, a small number of cases result from defects in BH4 metabolism (Blau, 2013; Blau et al., 2011) or from inactivating mutations in the molecular chaperone gene *DNAJC12* (Anikster et al., 2017). DNAJC12, in addition to BH4, facilitates proper folding of the PAH monomer (Blau, 2013; Straniero et al., 2017; Wortmann et al., 2013), improving its metabolic activity and, thus, indirectly improving phenylalanine-to-tyrosine metabolism.

### Treatment

Treatment of PKU relies on dietary management to control the amount of phenylalanine consumed in the diet (Vockley et al., 2014) to maintain blood plasma phenylalanine levels between 120 and 360 µmol/l. Practically, this means limiting natural protein intake and ingesting medical formulas and food that provide low-phenylalanine or phenylalanine-free synthetic protein while still containing other necessary nutrients and minerals. Metabolic control is monitored with frequent measurement of plasma amino acids to quantify phenylalanine and the phenylalanine-to-tyrosine ratio. The initiation of a phenylalanine-restricted diet soon after birth is essential to prevent the neurological, cognitive and behavioral sequelae of the biochemical perturbation (Gonzalez et al., 2011); however, the diet is difficult to maintain, especially in adolescent, young adult and pregnant patients. Dietary non-adherence results in neuropsychological issues, including anxiety and depression, which have been shown to have a lifelong impact on the quality of life (Brumm et al., 2010). Additionally, due to the significant fetal risks in untreated maternal PKU, achieving strict control of phenylalanine levels should be ideally initiated prior to conception or, if not possible, as early in the pregnancy as possible for optimal outcomes.

Another treatment approach stimulates residual PAH enzymatic activity by supplementation with a synthetic form of the cofactor BH4. Synthetic BH4 increases PAH activity in about 30% of patients (Ho and Christodoulou, 2014). In the responders, BH4 serves as a useful adjunct to the phenylalanine-restricted diet because it improves phenylalanine tolerance and may permit dietary liberation, thus increasing compliance (Keil et al., 2013). Although some *PAH* mutations are frequently seen in BH4-responsive patients (Kure and Shintaku, 2019), suggesting a genotype-phenotype correlation, there have been many exceptions. Patients with BH4 deficiencies (Hillert et al., 2020) and *DNAJC12* pathogenic variants can also respond to BH4 supplementation (Anikster et al., 2017; de Sain-van der Velden et al., 2018). Thus, all PKU patients, regardless of genotype, should be given a trial of synthetic BH4 (Vernon et al., 2010).

Although the main therapy for PKU focuses on diet modification and co-factor supplementation, another approach relies on converting accumulated phenylalanine to non-toxic metabolites. Phenylalanine ammonia lyase (PAL), which was initially discovered in plants (Levy et al., 2018), provides an alternative pathway for phenylalanine metabolism that coverts phenylalanine into ammonia and trans-cinnamic acid (Sarkissian et al., 1999). An injectable PEGylated version of PAL is now commercially available for PKU patients with phenylalanine levels >600 µmol/l (Thomas et al., 2018). This enzyme substitution therapy allows patients with PAH deficiency to achieve lower phenylalanine levels and potential liberalization of dietary management, although the immunogenicity to PAL and/or PEG are known adverse side-effect risks (Burton et al., 2020; Longo et al., 2018). There are efforts to further stabilize PAL, make the enzyme orally bioavailable and active, and reduce its immunogenicity (d'Amone et al., 2022), but this has not been translated to patients yet.

Although the pathophysiology of PKU is reasonably well understood and dietary management is effective in principle, patients still face a number of obstacles in their management of the disease, and therefore further research and new curative therapeutic options are still very much warranted to improve their prognosis and quality of life.

## Lysinuric protein intolerance

### Clinical features and epidemiology

Lysinuric protein intolerance (LPI), sometimes referred to as cationic aminoaciduria, is a rare and clinically variable disorder with features that include acute metabolic decompensation and pulmonary and immune involvement. Just over 200 patients with LPI have been reported in the literature. It was first described in the Finnish population by Perheentupa and Visakorpi (1965), and has an estimated prevalence of one in 60,000 in Finland and one in 52,000 in Northern Japan, but has also been reported in multiple different countries worldwide (Koizumi et al., 2003; Martinelli et al., 2020).

The clinical presentation of LPI ranges from acute neonatal hyperammonemia to chronic symptoms in adults. In its classic form, LPI can present in infancy after introduction of increased protein in the diet, either associated with a higher-protein-containing formula or protein-containing solid foods, resulting in postprandial hyperammonemia. Other early features can include feeding intolerance, vomiting or diarrhea (Alqarajeh et al., 2020). Older children may present with poor growth, hepatosplenomegaly, hypotonia and weakness, with easy bruising and fractures due to osteopenia. Patients often develop an aversion to protein-rich foods and adapt to vegetarian diets.

A literature review by Contreras et al. (2021) summarized previously published clinical features in 157 patients with LPI that were reported between 1990 and 2019. The most common symptoms were failure to thrive (52%), aversion to protein-rich food (36%), emesis (30%) and diarrhea (14.65%). Systemic manifestations spanned hematologic (55%), immune (54%), renal (41%), neurologic (25%) and pulmonary (18%) involvement, and multisystemic manifestations included hepatosplenomegaly (46%), proteinuria (34%), anemia (29%) and osteoporosis (24%). Neurologic impairment secondary to sequelae from repeated hyperammonemic episodes and seizures were also reported.

Between 66 and 71% of patients with LPI have lung involvement, with clinical findings ranging from asymptomatic interstitial lung disease identified by imaging to acute respiratory failure leading to death (Parto et al., 1993; Santamaria et al., 1996; Valimahamed-Mitha et al., 2015). The most life-threatening pulmonary complication is pulmonary alveolar proteinosis, which is characterized by the accumulation of surfactant in pulmonary alveoli and, in LPI, may occur due to impaired phagocytic activity of alveolar macrophages (Barilli et al., 2012). Pulmonary fibrosis is the second most notable pulmonary complication of LPI and may be found with or without alveolar proteinosis (Valimahamed-Mitha et al., 2015).

Renal complications from LPI can present with asymptomatic isolated proteinuria and mildly elevated creatinine preceding the eventual onset of renal failure. A retrospective review of 39 Finnish patients found proteinuria in 74%, hematuria in 38%, elevated blood pressure in 36% and end-stage renal disease in 10% of patients. Mild to moderate renal insufficiency developed in 59% of patients despite treatment (Tanner et al., 2007).

Hematologic and immune dysfunction are well-known complications of LPI, with a wide range of phenotypic presentations from variable cytopenias to hemophagocytic lymphohistiocytosis or macrophage activation syndrome. Cytopenias are fairly common and may include anemia, thrombocytopenia and leukopenia (Al-Samkari and Berliner, 2018). Immunologic manifestations have been reported in several cases and can manifest as systemic lupus erythematosus, rheumatoid arthritis, vitiligo and immune thrombocytopenic purpura (Aoki et al., 2001; Contreras et al., 2021; Kamoda et al., 1998; Mauhin et al., 2017; Parsons et al., 1996; Parto et al., 1993).

### Pathophysiology

LPI is caused by biallelic pathogenic variants in *SLC7A7*, which encodes the catalytic light chain of the heterodimeric amino acid transporter y+LAT1 (Borsani et al., 1999). The heavy chain of this transporter, 4F2hc, is encoded by *SLC3A2* (Fig. 1B). Together, these subunits form the cationic amino acid transport system y+L and transport the dibasic cationic amino acids lysine, arginine, and ornithine (Estevez et al., 1998). Reported pathogenic variants in *SLC7A7* have been loss-of-function, either by leading to nonsense-mediated mRNA decay, a truncated protein, loss of amino acid transport activity or protein mislocalization (Mykkanen et al., 2000; Rotoli et al., 2019; Sperandeo et al., 2005; Toivonen et al., 2002). However, no genotype-phenotype correlations have been found, and inter- and intra-familial clinical presentations vary even among individuals who are homozygous for the same variant (Sperandeo et al., 2000; Tringham et al., 2012).

The y+L cationic amino acid transport system is expressed in the placenta, platelets, skin fibroblasts, hepatocytes, the small intestine and the kidney (Wagner et al., 2001). In renal tubules and intestinal epithelia, the cationic amino acid transport system is located at the basolateral membrane and, via an antiport mechanism, couples the influx of sodium and neutral amino acids to the efflux of cationic amino acids into the bloodstream (Palacin et al., 2001). A defect in the y+L transport system reduces absorption of dibasic amino acids in the intestinal epithelia and decreases renal absorption of dibasic amino acids in the renal tubules. This leads to deficiencies in lysine, arginine and ornithine, as these amino acids are excreted in large amounts in urine. Deficiency of arginine and ornithine leads to a secondary urea cycle defect and hyperammonemia.

Dysregulation of nitric oxide (NO) production has been proposed as a potential mechanism for the multisystemic dysfunction in LPI, including lung, renal and immunologic involvement. The y+L system directs the efflux of cationic amino acids into the bloodstream, and its deficiency could potentially cause accumulation of arginine, which could induce superfluous NO synthesis. In support of this hypothesis, plasma 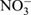 and citrulline were found to be increased in three patients, with fibroblasts from all three showing increased production of 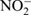 compared to fibroblasts from unaffected controls in the presence of fetal bovine serum, but this difference disappeared when the serum and non-essential amino acids were removed from the growth medium (Mannucci et al., 2005). Conversely, a subsequent study showed decreased NO in conditioned medium from cultured LPI patient-derived macrophages, suggesting intracellular arginine depletion. The same study found impaired Toll-like receptor (TLR) signaling, particularly via TLR9, downregulated type I interferons and overexpressed pro- and anti-inflammatory cytokines, which may instead explain the immune dysregulation in LPI (Kurko et al., 2015). More research is necessary to clearly elucidate the role of NO and the pathophysiology of immune dysregulation in LPI.

Studies in mouse models of LPI have proved difficult owing to the limited viability of homozygous *Slc7a7* mutant mice (Bodoy et al., 2019; Sperandeo et al., 2007; Stroup et al., 2020). However, a recently developed tamoxifen-inducible *Slc7a7* knockout mouse model successfully recapitulated several features of LPI. One month after tamoxifen induction, the knockout mice demonstrated a 50% reduction in survival compared to that of non-induced (*Slc7a7*-expressing) mice when placed on a low-protein diet (Bodoy et al., 2019). When the tamoxifen-induced knockout mice were supplemented with citrulline in addition to the low-protein diet, their survival reached 100% and they demonstrated improved body weight and food intake compared to those of tamoxifen-induced knockout mice on a low-protein diet alone. The tamoxifen-induced mice also exhibited brain edema, which was corrected by citrulline supplementation, and pulmonary alveolar proteinosis, which was reduced in incidence after treatment with citrulline (Bodoy et al., 2019).

### Diagnosis

The diagnosis of LPI is typically based on the combination of clinical symptoms and biochemical findings, confirmed by molecular genetic testing. Plasma amino acid measurements may reveal decreased concentrations of lysine, arginine and ornithine, although levels can be normal. Urine amino acid measurements may also reveal increased levels of these cationic amino acids, with lysine showing the greatest increase. Urine orotic acid levels may be elevated. Plasma glutamine, alanine, glycine, serine, proline and citrulline levels may also be elevated (Noguchi and Takahashi, 2019; Ogier de Baulny et al., 2012). Plasma ammonia levels are typically elevated after a protein-rich meal. Although biochemical analyses can aid in diagnosis, the absence of classical findings may not exclude the diagnosis, particularly in the setting of malnutrition.

Additional laboratory investigations may reveal findings consistent with hemophagocytic lymphohistiocytosis, although these are not specific to LPI, including elevated lactate dehydrogenase or ferritin, leukopenia, anemia, thrombocytopenia, fibrin abnormalities, elevated cholesterol or triglycerides, and mildly elevated liver transaminases (Noguchi and Takahashi, 2019; Ogier de Baulny et al., 2012).

The diagnosis of LPI may be challenging and is often delayed due to the non-specific nature of symptoms and the fact that biochemical readouts may be normal in the setting of protein restriction and malnutrition (Alqarajeh et al., 2020). A high index of suspicion is necessary, and molecular testing demonstrating two pathogenic variants in the *SLC7A7* gene confirms the diagnosis. The pathogenic variants in *SLC7A7* reported so far have been distributed along the entire gene (Sperandeo et al., 2008). Founder mutations have been identified in Finland (c.895-2A>T, p.Tyr299Ilefs*10), Japan (p.R410X) and Italy (c.1384_1385insACTA) (Sebastio et al., 2011).

Although LPI is not one of the conditions included in NBS panels, it has been diagnosed after abnormal NBS results in two published cases, following the detection of mild elevations in citrulline (Siri et al., 2022; Yahyaoui et al., 2020). This demonstrates that, despite its rarity, it is important to consider LPI in the differential diagnosis when NBS identifies elevated citrulline, given that symptoms are highly variable.

### Treatment

Treatment of LPI aims to prevent hyperammonemia while providing enough natural protein and essential amino acids for appropriate growth. Dietary protein intake is limited to 1-1.5 g/kg/day in children and 0.5-0.8 g/kg/day in adult patients (Ogier de Baulny et al., 2012). Citrulline supplementation is used to prevent postprandial hyperammonemia and to increase protein tolerance by urea cycle intermediates; a conservative dose of <100 mg/kg/day in four divided doses is recommended, given that large amounts of citrulline are partially converted into arginine intracellularly and in light of the theory that intracellular accumulation of arginine may lead to the dysregulation of NO synthesis (Ogier de Baulny et al., 2012). To keep plasma lysine in the normal range, lysine should be supplemented at low doses of 10-40 mg/kg/day (Sebastio et al., 2011). Carnitine supplementation of 20-–50 mg/kg/day is recommended to correct secondary hypocarnitinemia, if present (Tanner et al., 2008).

The effectiveness of diet modification and supplementation should be routinely monitored by measuring plasma ammonia and glutamine levels and urinary orotic acid levels. In patients with persistent hyperammonemia, despite adherence to dietary modifications and supplementation, a more restrictive protein intake and use of nitrogen scavengers should be considered (Sebastio et al., 2011). Treatment of non-metabolic manifestations should be managed as per established guidelines and is not specific to LPI; for example, growth hormone may be administered to patients with impaired endogenous secretion, and bronchoalveolar lavage steroids and immunosuppressors may be required for patients with severe alveolar proteinosis.

Further studies and development of improved models are necessary to fully investigate the pathophysiology of how LPI causes such a severe systemic phenotype. Although the secondary urea cycle defects in LPI are generally understood and treated in a targeted manner, the pathophysiological mechanisms of growth failure, osteopenia, renal hematology and immune dysfunction have not been elucidated.

## Homocystinuria due to CBS deficiency

### Clinical features and epidemiology

Homocystinuria is a group of hereditary disorders of homocysteine (Hcy) metabolism characterized by elevated Hcy levels, typically associated with increased risk of thromboembolism and neurocognitive features (Carson et al., 1963; Gerrard and Dawson, 2022; Mudd et al., 1964). Individuals with classical homocystinuria due to CBS deficiency appear clinically normal at birth, and clinical features can present at any age and with a high degree of variability. The NBS data-based estimated incidence of classic homocystinuria worldwide is one in 200,000 to one in 340,000, although this is likely an underestimate owing to current limitations in detection by NBS and clinical ascertainment (Sellos-Moura et al., 2020; Skovby et al., 2010). The prevalence varies among populations, with the highest known prevalence in Qatar (one in 1800) (Gan-Schreier et al., 2010). Homocystinuria is thought to have a prevalence of one in 6400 in Norway and one in 65,000 in Ireland (Gan-Schreier et al., 2010; Moat et al., 2004; Naughten, Yap, and Mayne, 1998; Refsum et al., 2004).

Approximately 60% of affected individuals show developmental delay and cognitive impairment, and about 50% of untreated individuals are reported to have seizures and/or neuropsychiatric manifestations such as anxiety, depression or psychosis (Yap et al., 2001b). Lens dislocation (ectopia lentis) is typically seen in most untreated individuals by the age of 5-10 years, and invariably by the fourth decade (Mulvihill et al., 2001). Ectopia lentis is considered one of the most consistent clinical findings in classic homocystinuria and results from disruption of the zonular lens fibers (Mudd et al., 1985). Individuals with homocystinuria typically have tall stature with arachnodactyly and may show elongation of long bones (dolichostenomelia) at the time of puberty, in addition to scoliosis and pectus excavatum (Brenton, 1977). These skeletal abnormalities often lead to a Marfanoid appearance but, unlike individuals with Marfan syndrome, patients with homocystinuria often show restricted joint mobility. Affected adults often have osteopenia or osteoporosis, with up to 50% showing osteoporosis by their teens (Weber et al., 2016). Based on population-based studies, elevated Hcy has also been suggested to be an independent risk factor for osteoporotic fractures (van Meurs et al., 2004).

Arterial and venous thromboembolism, often cerebrovascular, is a major cause of morbidity in individuals with homocystinuria and can affect any vessel. Thromboembolic events typically emerge in young adults, although these have also been seen in infants (Karaca et al., 2014; Kelly et al., 2003; Magner et al., 2011; Yap, 2003; Yap et al., 2001a; Harker et al., 1974; Perła-Kaján et al., 2007). To mitigate the morbidity of the vascular phenotypes, individuals with homocystinuria should avoid oral contraceptives, and prophylactic anticoagulation is recommended during the third trimester of pregnancy and briefly postpartum (Pierre et al., 2006). Surgery for any individual with homocystinuria should be undertaken with caution due to high risk of thromboembolism.

### Pathophysiology

Elevated Hcy levels are seen in cobalamin (vitamin B12) and folate deficiency, in chronic kidney disease due to impaired renal excretion of Hcy, in exposure to some drugs/toxins such as nitrous oxide or methotrexate, or as a result of pathogenic variants in genes encoding enzymes involved in cobalamin metabolism, methionine metabolism or remethylation. The focus of this discussion will be on classic homocystinuria, which results from pathogenic variants in *CBS*, the gene encoding cystathionine β-synthase (CBS). It is important to note that pathogenic variants in methyltetrahydrofolate reductase (*MTHFR*) and disorders of cobalamin metabolism can also lead to elevated Hcy levels, although with some distinct biochemical and clinical features compared to those of classic homocystinuria (Gerrard and Dawson, 2022; Sloan et al., 1993). Additionally, specific pathogenic variants in *CBS* result in defective S-adenosylmethionine (SAM) regulation of CBS. Clinically, this correlates to a clinical subtype with partial pyridoxine responsiveness, reduced risk for connective tissue manifestations and ongoing thrombosis risk (Kluijtmans et al., 1996; Maclean et al., 2002).

Classic homocystinuria was first described in individuals with neurocognitive and Marfanoid features who were found to have increased urinary excretion of methionine and Hcy, and liver biopsies later revealed a lack of CBS activity (Carson et al., 1963; Mudd et al., 1964). Hcy is synthesized from methionine in the transsulfuration pathway and is subsequently metabolized to cystathionine and then cysteine via CBS and cystathionine γ-lyase (CTH), respectively (Fig. 1C). Pathogenic variants affecting CBS function lead to Hcy accumulation in both blood and urine, termed homocystinemia and homocystinuria, respectively (Gerrard and Dawson, 2022). S-adenosylhomocysteine (SAH), SAM and methionine also accumulate, along with decreased levels of cystathionine and cysteine.

The molecular events that lead to the vascular phenotypes seen in homocystinuria are not entirely clear, but Hcy is believed to cause direct injury to endothelial cells and platelets via free radical formation, leading to increased risk of thrombosis. In addition, protein modification via homocysteinylation is suggested to play a role in atherogenesis (Harker et al., 1974; Perła-Kaján et al., 2007). Skeletal and ocular features seen in individuals with homocystinuria have been proposed to result from abnormal modification of sulfhydryl groups, causing impaired cross-link formation in proteins such as collagen and elastin. Altered regulation of lysyl oxidase and increased oxidative stress may also contribute (Kang and Trelstad, 1973; Lubec et al., 1996; Saito and Marumo, 2018). Homocysteinylation of fibrillin-1 (FBN1) and fibronectin (FN1) have similarly been suggested to impair extracellular matrix function and organization (Saito and Marumo, 2018).

### Diagnosis

Classic homocystinuria is included in NBS and is tested for via tandem mass spectrometry-based measurement of methionine in dried blood spots from infants. Elevated methionine levels from 200 to 1500 µM (reference 10-40 µM) indicate a positive screening result. If the threshold methionine level is exceeded on the initial NBS, a repeat dried blood spot may be evaluated or quantitative plasma amino acid analysis and total plasma Hcy levels may be directly obtained. Of note, infants with pyridoxine (vitamin B6)-responsive homocystinuria are rarely identified via NBS, presumably because methionine levels do not yet reach the screening threshold in this patient subtype at the time the NBS is performed (Levy, 2021a). This is thought to contribute to incomplete ascertainment and to the likelihood that homocystinuria may be more prevalent than currently appreciated. As discussed above, Qatar has the highest reported incidence of homocystinuria and includes direct measurement of Hcy in its NBS program (Gan-Schreier et al., 2010). However, direct Hcy measurements are not performed as a universal screening approach in other countries due to the need for samples to be chemically reduced to measure Hcy (more details below) (Gerrard and Dawson, 2022).

Regarding biochemical analysis, classic homocystinuria is characterized by elevated plasma Hcy and methionine, as well as decreased cysteine, as these are distal to the impaired CBS. Sample preparation with reduction of thiol bonds allows for quantification of total Hcy, typically via high-performance liquid chromatography, mass spectrometry or immunoassays. Total Hcy levels are typically measured from whole-blood samples, ideally transported on ice because blood cells can release Hcy at room temperature, and are not affected by fasting status (Gerrard and Dawson, 2022). Complete blood count, vitamin B12 and folate levels, plasma amino acid levels and assessment of kidney function can also be valuable to evaluate whether the elevated Hcy levels represent a diagnosis of classic homocystinuria or other etiologies, such as renal disease, vitamin B12 or folate deficiency, MTHFR deficiency, or a disorder of intracellular cobalamin metabolism. Guidelines for diagnosis of homocystinuria include measurement of plasma amino acid profiles and total Hcy levels. Total plasma Hcy levels exceeding 100 µM in the setting of high methionine levels are highly suggestive.

Regardless of diagnostic challenges, homocystinuria should be considered in any individual who presents with developmental delay, neurological symptoms such as seizures or movement disorder, unprovoked thromboembolism, and high myopia or ectopia lentis. Risk of thromboembolism appears to be proportional to the degree and duration of Hcy elevation, with risk of a thromboembolic event rising to 50% by the age of 30 years in untreated individuals (Mudd et al., 1985). Ectopia lentis can occur within the first decade of life in ∼70% of untreated individuals with homocystinuria and may lead to insidious loss of visual acuity if not detected and treated (Mudd et al., 1985).

Classic homocystinuria shows autosomal recessive inheritance and is caused by biallelic pathogenic variants in *CBS* (Miles and Kraus, 2004). More than 160 pathogenic mutations are currently known, the majority of which are private missense mutations, i.e. they are found in individuals or single families (Kraus et al., 1999; Sacharow, Picker, and Levy, 1993). The p.Ile278Thr and p.Gly307Ser variants in exon 8 are the most common among many populations (Gan-Schreier et al., 2010), although the p.Arg336Cys variant accounts for 93% of affected individuals of Qatari ancestry. Overall, >95% of classic homocystinuria cases can be detected by single-gene sequencing, with the remainder detectable by gene-targeted deletion/duplication analysis (Sacharow et al., 1993). CBS enzyme activity analysis in fibroblasts or plasma can be performed in instances in which clinical suspicion is high but genetic testing does not confirm a molecular diagnosis.

### Treatment

The goal of treatment for classic homocystinuria is to achieve normal plasma levels of free Hcy (<11 µM), and a plasma level of total Hcy <100 µM in pyridoxine non-responsive patients and <50 µM in pyridoxine-responsive patients (Morris et al., 2017). There are several strategies to accomplish this. Because CBS uses pyridoxal 5′-phosphate as a cofactor, normal Hcy levels can be achieved in some affected individuals through supplementation with high-dose pyridoxine, a pyridoxal phosphate (PLP) precursor. A phenotypic spectrum of pyridoxine responsiveness has been described, with non-responders typically showing severe clinical features in childhood when untreated and extreme responders typically presenting with thromboembolism in adulthood without other phenotypic features (Kožich et al., 2021). Intuitively, pyridoxine responsiveness appears to be most relevant in individuals with genetic variants that affect the PLP cofactor-binding site in CBS. The common p.Ile278Thr allele often predicts pyridoxine responsiveness, whereas the common p.Gly307Ser allele predicts non-responsiveness (Gaustadnes et al., 2002; Skovby et al., 2010). Pyridoxine responsiveness can be evaluated by administering high-dose pyridoxine (100-500 mg daily) and measuring total Hcy levels over the course of 1 week, with >30% reduction in Hcy suggestive of pyridoxine responsiveness (Morris et al., 2017). The goal is to administer the lowest pyridoxine dose that still provides an effect, as persistent high doses of >900 mg/day carry risk of peripheral neuropathy.

The (re)methylation of Hcy to methionine, another strategy to lower plasma Hcy, can be enhanced by supplementing patients with the methyl donor betaine (N,N,N-trimethylglycine), which provides a substrate for the methyltransferase. Although it helps to clear Hcy, this treatment elevates methionine levels. This poses a health risk, as methionine levels of >500 µM can be associated with cerebral edema (Braverman et al., 2005; Devlin et al., 2004; Schwahn et al., 2020; Yaghmai et al., 2002).

Conversely, most individuals with homocystinuria require restricted intake of methionine, as this amino acid is the precursor to Hcy synthesis. This is achieved by limiting natural protein intake and supplementing with methionine-free amino acid formulas to prevent protein malnutrition and insufficiency of other amino acids. Folate and cobalamin supplementation is recommended to enhance Hcy remethytlation to methionine. Cysteine should be supplemented if it is low.

Homocystinuria is a distinct example of an inborn error in metabolism with pleiotropic systemic manifestations that remain incompletely understood in relation to the specific metabolic defect, but are modifiable in the majority of affected individuals through dietary interventions.

## Advances in treatment – beyond cofactor and substrate management

Dietary management to correct the biochemical defects associated with PKU, including restriction of phenylalanine and repletion of tyrosine, is effective in remediating the amino acid abnormalities and manifestations of the disease. However, this is extremely difficult to adhere to and thus not a reasonable ‘real-world’ therapy for many patients. Fortunately, introduction of both synthetic BH4 for cofactor management and PEGylated PAL for enzyme replacement (ERT) has alleviated some of the burden of strict dietary management for many patients with PKU. Similar to PKU, dietary management in classic homocystinuria can significantly remediate the abnormal accumulation of pathogenic amino precursors, but this management is extremely onerous and difficult to adhere to. Thus, other therapeutic approaches are necessary.

Beyond dietary therapy, enzyme, gene and mRNA replacement are approaches for effective treatment of these disorders. As discussed above, ERT has already entered the clinic for PKU. Moreover, ERT using PEGylated CBS has been studied in a mouse model of classic homocystinuria and achieved ∼75% reduction in Hcy and normalization of cysteine levels (Bublil and Majtan, 2020; Bublil et al., 2016). A clinical trial extending from these studies using a modified version of CBS (pegtibatinase) is ongoing (NCT03406611), as is an early-stage clinical trial involving the use of pegtarviliase, a recombinant enzyme with the capacity to degrade Hcy (NCT05154890).

Gene therapy to provide exogenous PAH has been proposed and tested in mouse models of PKU since the 1990s (Eisensmith and Woo, 1994). Adeno-associated virus (AAV)-based gene delivery is just now in phase 1/2 studies (NCT03952156 and NCT04480567) (Ahmed et al., 2020; Grisch-Chan et al., 2019). Other methods of PAH correction using base-editing approaches (Villiger et al., 2018) are currently in pre-clinical development. AAV-based gene therapy to deliver a human *CBS* cDNA has been explored in mouse models of classic homocystinuria and showed dose-dependent increase in hepatic CBS activity, a promising reduction in serum Hcy levels and amelioration of disease-associated phenotypes (Lee et al., 2021). Licensing efforts for human clinical trials are reportedly underway (Majtan et al., 2023).

Additionally, compensating for an enzyme deficiency by providing mRNA via encapsulated lipid nanoparticles is being studied in clinical trial settings for a number of other inborn errors of metabolism (An et al., 2018; Balakrishnan et al., 2020; Zhu et al., 2019). This approach is being evaluated in preclinical settings for PAH deficiency (Diaz-Trelles et al., 2022; Diaz-Trelles and Perez-Garcia, 2022).

Two other approaches under consideration for the treatment of classic homocystinuria include chaperone therapy and modification of the gastrointestinal microbiome. Because the majority of pathogenic CBS variants are missense, which are believed to lead to protein misfolding, conformational correction via chaperones may be a feasible treatment strategy for classic homocystinuria, although no candidates have yet been identified (Majtan et al., 2023; Singh et al., 2007). In another interesting approach, a genetically engineered strain of probiotic *Escherichia coli* that can metabolize methionine within the gastrointestinal system has been developed and is now in phase I clinical trials (Majtan et al., 2023). Bacterial metabolism of methionine would prevent its uptake and conversion to Hcy; using a similar principle, recombinant enzymes capable of degrading methionine in the gastrointestinal tract or bloodstream are also in development (Majtan et al., 2023).

Each of these treatment approaches has benefits and drawbacks, and eventually it may be possible for patients to choose from a wide variety of treatment options taking into consideration dosing frequency, i.e. regular dosing in ERT or mRNA therapy versus possible one-time dosing for AAV-mediated gene therapy; side effects such as risk of anaphylactic reactions or infusion/injection reactions in ERT; treatment cost/availability; and effectiveness, i.e. partial versus complete responses in cofactor, dietary or chaperone therapy or a combination of therapies. In fact, with the availability of dietary, cofactor and ERT therapies, this individualized disease-specific approach is already becoming a reality for PKU.

## Conclusions

In conclusion, disorders of amino acid metabolism are a diverse group of genetic conditions that have profound and wide-ranging effects on the health of affected individuals. This diversity extends beyond individual genetic etiologies, and encompasses the age of onset, severity and clinical manifestations, which can vary widely from one individual disorder to another. From PKU to homocystinuria to LPI, each disorder offers unique challenges and necessitates tailored treatment approaches.

Despite the often-daunting challenges posed by these disorders, advances in research have paved the way for a better understanding of the natural history of each disorder, novel approaches to treatment and improved management strategies.
